# Epidemiology and genomics of azithromycin-resistant extensively drug-resistant *Salmonella enterica* serovar Typhimurium in Taiwan

**DOI:** 10.1128/aac.01334-25

**Published:** 2026-02-19

**Authors:** Yu-Ping Hong, Ying-Shu Liao, Bo-Han Chen, You-Wun Wang, Ru-Hsiou Teng, Shiu-Yun Liang, Hsiao Lun Wei, Jui-Hsien Chang, Chien-Shun Chiou

**Affiliations:** 1 Center for Research, Diagnostics and Vaccine Development, Centers for Disease Control, Ministry of Health and Welfare, Taichung, Taiwan; Shionogi Inc., Florham Park, New Jersey, USA

**Keywords:** non-typhoidal *Salmonella *(NTS), antimicrobial resistance, multidrug-resistant (MDR), extensively drug-resistant (XDR), azithromycin, resistance mechanism, molecular epidemiology, IncC plasmid

## Abstract

The recent emergence of azithromycin-resistant extensively drug-resistant (AziR-XDR) *Salmonella enterica* serovar Typhimurium (*S*. Typhimurium) in Taiwan poses a significant public health concern. To investigate the genetic basis and the evolutionary dynamics, we analyzed 60 isolates collected between 2007 and 2024, comprising multidrug-resistant (MDR, *n* = 45) and AziR-XDR (*n* = 15) isolates from human and animal sources. Whole-genome sequencing analysis revealed that 36 of 45 MDR isolates and all AziR-XDR isolates belonged to the HC50_13 subclone (differing by ≤ 50 core genes) and clustered within the HC20_152604 cluster (differing by ≤ 20 core genes) and the SNP cluster PDS000042202. All HC50_13 MDR isolates and AziR-XDR isolates carried core resistance genes including *aac(3)-IId*, *aph(3’’)-Ib*, *aph(6)-Id*, *blaTEM-1*, *floR*, *sul2*, *tet(A*) on type 2 IncC plasmids, and AziR-XDR isolates carried additional antimicrobial resistance genes (ARGs) on the plasmids, including *blaDHA-1*, *dfrA17*, *mph(A*), *qnrB*, and *sul1*, conferring additional resistance to azithromycin, trimethoprim, third-generation cephalosporins, and fluoroquinolones. AziR-XDR *S. Typhimurium* strains belonging to the HC50_13 subclone were initially identified in 2016 and have notably increased in Taiwan since 2021. The subclone was likely evolved from MDR ancestor strains by multiple IS*26*-mediated acquisitions of additional resistance cassettes. Our findings demonstrate how MDR *S*. Typhimurium can evolve locally into highly resistant strains. This underscores the need for genomic surveillance and coordinated antimicrobial stewardship in Taiwan and globally to prevent further dissemination of the highly resistant HC20_152604 clade.

## INTRODUCTION

*Salmonella enterica* serovar Typhimurium (*S*. Typhimurium) is a major zoonotic pathogen causing gastrointestinal and invasive infections worldwide. In Taiwan, *S*. Typhimurium has consistently ranked as the second most common non-typhoidal *Salmonella* (NTS) serovar responsible for human infections following *S*. Enteritidis (1). Alarmingly, *S*. Typhimurium isolates in Taiwan have shown substantially higher levels of antimicrobial resistance compared with those in many Western countries, such as Denmark (2). Multidrug-resistant (MDR) strains, defined as resistant to at least three different classes of antimicrobials, are prevalent in human and animal reservoirs (3).

Invasive *S. Typhi* infections have become increasingly difficult to treat due to the global spread of MDR and XDR strains (4–6). With resistance emerging to the traditional first-line antimicrobials (ampicillin, chloramphenicol, trimethoprim-sulfamethoxazole), fluoroquinolones, and third-generation cephalosporins, azithromycin has become one of the few remaining effective options; notably, it succeeded where carbapenem therapy failed in the only reported XDR *S. Typhi* case in Taiwan (7). Azithromycin has increasingly been used to treat invasive NTS infections (4); resistance has also escalated. In Taiwan, *Salmonella* has been found to harbor diverse azithromycin resistance mechanisms, including plasmid-borne *erm(B*), plasmid- and chromosome-borne *mph(A*), plasmid- and chromosome-borne *erm(42*), and efflux pump activation (8). The growing prevalence and diversity of these resistance determinants highlight the urgent need for sustained surveillance and molecular characterization to guide treatment and containment.

Whole-genome sequencing (WGS) has fundamentally changed bacterial genomics and clinical microbiology (9). It enables simultaneous pathogen identification, high-resolution analysis of population structure and evolutionary dynamics, and detection of antimicrobial resistance and virulence determinants. The routine use of WGS in bacterial surveillance has highlighted the need for scalable and standardized analytical schemes to translate sequencing data into comparable genotypic information across laboratories and data sets. The core genome multilocus sequence typing (cgMLST) method analyzes WGS data to generate standardized allelic profiles, enabling results to be compared across laboratories without sharing raw data. In *Salmonella*, the hierarchical clustering of cgMLST (HierCC) approach further organizes isolates into discrete, nested clusters at multiple genetic distance levels (10). Each cluster assignment provides an easily interpretable measure of relatedness between strains, eliminating the need for routine phylogenetic tree reconstruction.

In Taiwan, HierCC has been applied to investigate long-term population shifts of *S*. Typhimurium. An analysis of isolates collected between 2004 and 2019 showed that most strains belonged to seven major HC100 clones, which differ by more than 100 core genes (11). Among these, HC100_13 was particularly notable. It is further divided into two HC50 subclones, defined as groups differing by 50 or fewer core genes, namely HC50_13 and HC50_6770, which were associated with distinct resistance profiles and plasmid types (11). These findings highlight the utility of HierCC in resolving the genomic population structure of *S*. Typhimurium and provide a framework for monitoring the emergence of highly resistant lineages in Taiwan. In parallel, the NCBI Pathogen Detection system organizes genomes into SNP clusters. These serve as single-level categories of closely related isolates and are useful for broad genomic comparisons, but they lack the hierarchical resolution offered by HierCC.

In 2023–2024, an outbreak of azithromycin-resistant extensively drug-resistant (AziR-XDR) *S*. Typhimurium was detected in central and southern Taiwan. These strains posed a serious therapeutic challenge because they were resistant to all traditional first-line antimicrobials, fluoroquinolones, third-generation cephalosporins, and azithromycin. In this study, we investigated the epidemiological trends and genomic features of AziR-XDR isolates in the context of local *S*. Typhimurium population structure. Using WGS-based approaches, we focused on the resistance determinants, their plasmid backbones, and the phylogenetic relationships that define this emerging highly resistant clade. Together, these analyses provide insights into how MDR *S*. Typhimurium has evolved locally into highly resistant lineages and highlight the implications for surveillance and containment strategies in Taiwan.

## RESULTS

### Epidemiology of Azi-XDR *S*. Typhimurium

The HC100_13 clone represents one of the major *S*. Typhimurium lineages, identified by PFGE pattern clustering and confirmed by WGS-based HierCC analysis as previously described (11), defined as isolates differing by ≤100 core genes. Overall, HC100_13 clone accounted for 9.0% of all *S*. Typhimurium isolates collected between 2004 and 2024 (Table S1). Although this clone was rarely detected before 2014, its frequency increased substantially in subsequent years. Within the HC100_13 cluster, a distinct sublineage emerged that exhibited reduced susceptibility to ciprofloxacin and resistance to multiple antimicrobial classes, including ampicillin, azithromycin, third-generation cephalosporins (cefotaxime and ceftazidime), chloramphenicol, gentamicin, sulfamethoxazole, trimethoprim, and tetracycline. Isolates belonging to this sublineage were designated as AziR-XDR.

**Fig 1:**
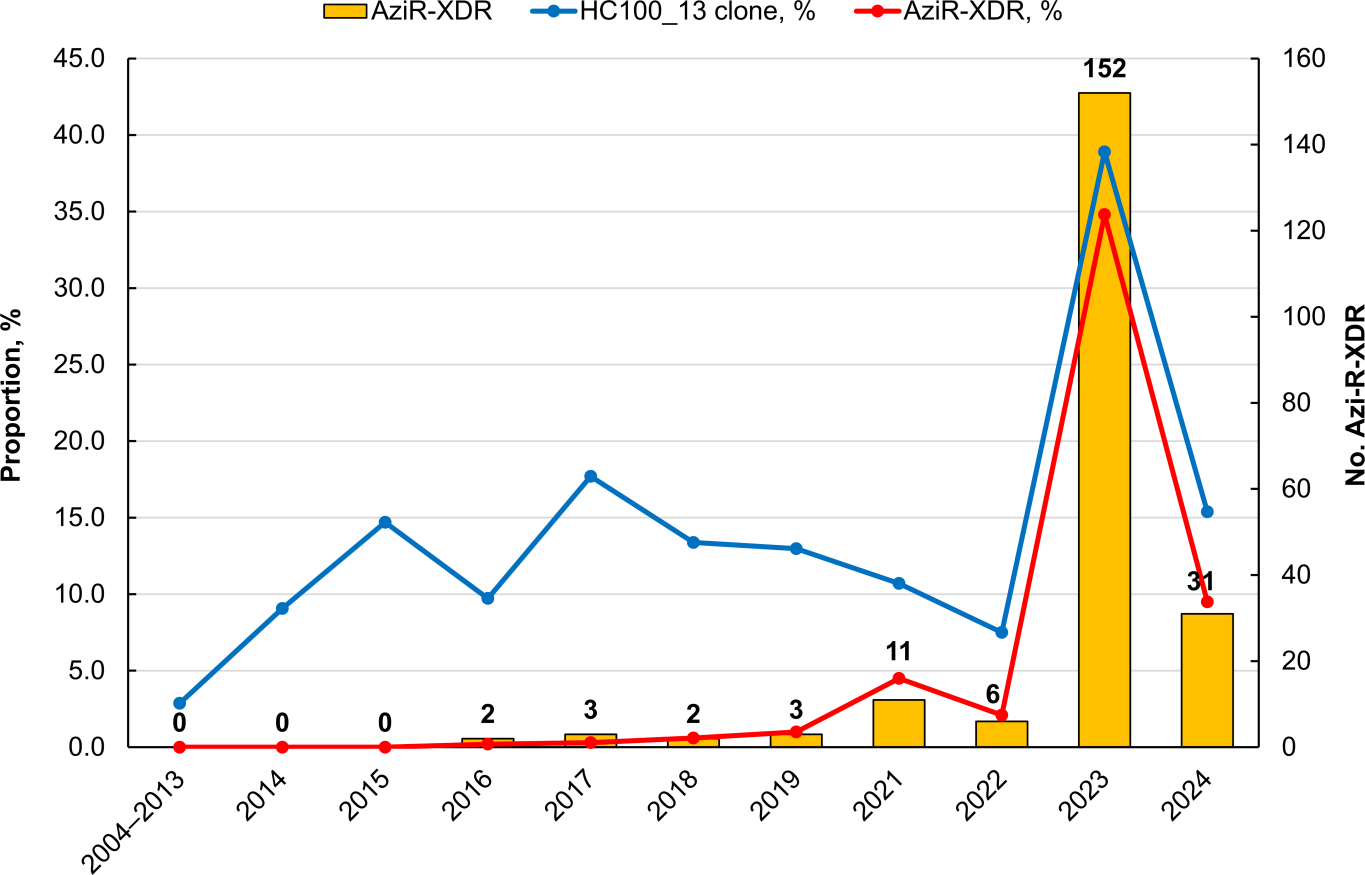
Temporal trends of *Salmonella enterica* serovar Typhimurium HC100_13 clone and AziR-XDR strains in Taiwan, 2004–2024. The number of AziR-XDR *S*. Typhimurium isolates for each year is shown. Detailed numerical data are summarized in Table S1.

AziR-XDR *S*. Typhimurium strains were first identified in 2016 and showed a sharp rise in 2023 (Fig. 1). This marked increase was primarily driven by the expansion of the AziR-XDR sublineage within HC100_13. HC100_13 accounted for 38.9% (170/437) of all *S*. Typhimurium isolates in 2023, of which 34.8% (152/437) were AziR-XDR (Table S1).

Extraintestinal infection rates were compared among seven major *S*. Typhimurium HC100 clones, HC100_2, HC100_13, HC100_41, HC100_310, HC100_305, HC100_501, and HC100_46261, based on isolates collected in Taiwan between 2004 and 2024 (Table S2). Chi-square analysis indicated significant differences among clones (*P* < 0.0001), with particularly high rates observed in HC100_501 (36.82%) and HC100_305 (20.37%). The extraintestinal infection rate for HC100_13 was 5.43%. Within this clone, there was no significant difference in extraintestinal infection rates between AziR-XDR and other HC100_13 isolates (*P* = 0.1811). Further analysis also showed no significant differences between the two groups in sex distribution (*P* = 0.8806) or age group distribution (*P* = 0.9513).

### Genomic characteristics of MDR and AziR-XDR *S*. Typhimurium strains

To investigate the phylogeny, ARGs, and associated mobile genetic elements, we conducted whole-genome sequencing of 45 MDR and 15 AziR-XDR *S*. Typhimurium isolates. Enterobase HierCC assignments showed that the isolates belonged to two subclones, HC50_13 (*n* = 51) and HC50_6770 (*n* = 9) (Table S3). These two subclones displayed distinct resistance and plasmid profiles. Within each subclone, however, isolates were highly clonal. All HC50_13 isolates belonged to the same HC20 cluster (HC20_152604), with 47 further grouped into HC10_152604, while all HC50_6770 isolates fell within the same HC10 cluster (HC10_32544). The NCBI Pathogen Detection pipeline assigned all HC50_13 isolates to SNP cluster PDS000042202, and HC50_6770 isolates to cluster PDS000042407.

The two HC50 subclones displayed distinct plasmid profiles. All HC50_13 isolates carried type 2 IncC plasmids, whereas all HC50_6770 isolates harbored plasmids with IncFIA(HI1), IncHI1A, IncHI1B(R27), and IncQ1 replicons (Table S4). HC50_13 isolates carried 7–19 ARGs, while HC50_6770 isolates carried 8–12 ARGs (Table S3). Among the HC50_13 isolates, nine ARGs, *aac(3)-IId*, *aph(6)-Id*, *blaTEM-1*, *sul2*, *tet(A*), *floR*, *sul1*, *blaDHA-1*, and *aph(3’’)-Ib*, were detected in 90.2%–100% of isolates (Table S5). In contrast, all HC50_6770 isolates carried seven core ARGs, including *aadA2*, *aph(3’’)-Ib*, *aph(3’)-Ia*, *aph(6)-Id*, *dfrA12*, *sul2*, and *tet(B*), with most (8 of 9) additionally harboring *aac(3)-IId*, *blaTEM-1*, and *bleO*.

### Complete genome assemblies and associated ARG-carrying plasmids

To define the genomic context of ARGs, we generated complete genomes for 25 (23 HC50_13 and 2 HC50_6770) isolates using ONT sequencing. Assemblies showed that ARGs were borne on plasmids with diverse replicon profiles (Table 1). All HC50_13 isolates carried type 2 IncC plasmids harboring 7–14 ARGs. By contrast, both HC50_6770 isolates carried a hybrid plasmid with IncFIA(HI1)–IncHI1A–IncHI1B(R27)–IncQ1 replicons and 10 ARGs. Additional hybrid plasmids were also found among HC50_13 isolates, including IncHI2–IncHI2A, IncHI1A–IncHI1B(R27), and IncFIA(HI1)–IncHI1A–IncHI1B(R27), each harboring 7–9 ARGs. IncI1-I(α) plasmids were associated with *blaCMY-2* and macrolide resistance genes *erm(B*) and *erm(42*); IncFIB(pHCM2) plasmids carried *dfrA51*; IncN plasmids carried *blaCTX-M-65*; and IncB/O/K/Z plasmids carried *blaCTX-M-14* and *erm(B*).

**TABLE 1:** Complete genome assemblies and ARG-carrying plasmids in 2 HC50_6770 and 23 HC100_13 *Salmonella enterica* serovar Typhimurium isolates from Taiwan, 2007–2024^*a*^

IsolateID	Year	AssemblyID	Length (bp)	Replicon	ARGs	MDR gene cluster
CC07.136	2007	CC07.136_chr	4,790,990			
		pCC07.136_234k	234,102	IncFIA(HI1)-IncHI1A-IncHI1B(R27)-IncQ1	*aac(3)-IId, aadA2, aph(3'')-Ib, aph(3')-Ia, aph(6)-Id, blaTEM, bleO, dfrA12, sul2, tet(B*)	
		pCC07.136_87k	87,749	IncB/O/K/Z	*blaCTX-M-14, erm(B*)	
NL08.094	2008	NL08.094_chr	4,790,990			
		pNL08.094_234k	234,102	IncFIA(HI1)-IncHI1A-IncHI1B(R27)-IncQ1	*aac(3)-IId, aadA2, aph(3'')-Ib, aph(3')-Ia, aph(6)-Id, blaTEM, bleO* (partial)*, dfrA12, sul2, tet(B*)	
D151	2012	D151_chr	4,788,170			
		pD151_158k	158,762	IncC	*aac(3)-IId, aadA2, aph(3'')-Ib, aph(3')-Ia, aph(6)-Id, blaDHA-1, blaTEM-1, floR, sul1, sul2, tet(A*)	A2
D154	2012	D154_chr	4,790,089			
		pD154_158k	158,762	IncC	*aac(3)-IId, aadA2, aph(3'')-Ib, aph(3')-Ia, aph(6)-Id, blaDHA-1, blaTEM-1, floR, sul1, sul2, tet(A*)	A2
R16.0248	2014	R16.0248_chr	4,792,713			
		pR16.0248_193k	193,757	IncHI1A-IncHI1B(R27)	*aadA1, aadA2, aadA22, blaTEM-1, bleO* (partial)*, dfrA12, floR, lnu(F), qnrS1, sul3*	
		pR16.0248_152k	152,329	IncC	*aac(3)-IId, aadA2, aph(3'')-Ib, aph(3')-Ia, aph(6)-Id, blaTEM-1, floR, sul1, sul2, tet(A*)	A1
		pR16.0248_41k	41,725	IncN	*blaCTX-M-65*	
R16.0567	2014	R16.0567_chr	4,827,249			
		pR16.0567_275k	275,789	IncHI2-IncHI2A	*aac(3)-IId, aac(3)-IVa, aadA2, aph(4)-Ia, bleO* (partial)*, dfrA12, lnu(F), mcr-9.1*	
		pR16.0567_158k	158,762	IncC	*aac(3)-IId, aadA2, aph(3'')-Ib, aph(3')-Ia, aph(6)-Id, blaDHA-1, blaTEM-1, floR, sul1, sul2, tet(A*)	A2
SD14.124 (R14.0938)	2014	SD14.124_chr	4,790,064			
		pSD14.124_207k	207,551	IncFIA(HI1)-IncHI1A-IncHI1B(R27)	*aadA1, aadA2, aadA22, blaTEM-1, bleO* (partial)*, dfrA12, floR, lnu(F), qnrS1, sul3*	
		pSD14.124_185k	185,938	IncC	*aac(3)-IId, aadA2, aph(3'')-Ib, aph(3')-Ia, aph(6)-Id, blaDHA-1, blaTEM-1, floR, sul1, sul2, tet(A*)	A2
R15.0455	2015	R15.0455_chr	4,792,679			
		pR15.0455_142k	142,637	IncC	*aac(3)-IId, aph(3'')-Ib, aph(6)-Id, blaTEM-1, floR, sul2, tet(A*)	O
R16.1923	2016	R16.1923_chr	4,790,128			
		pR16.1923_170k	170,704	IncC	*aac(3)-IId, aadA2, aph(3'')-Ib, aph(3')-Ia, aph(6)-Id, blaDHA-1, blaTEM-1, dfrA17, floR, mph(A), qnrB, sul1, sul2, tet(A*)	A4
		pR16.1923_100k	100,140	IncI1-I(Alpha)	*blaCMY-2*	
R16.2845	2016	R16.2845_chr	4,767,087			
		pR16.2845_161k	161,012	IncC	*aac(3)-IId, aph(3'')-Ib, aph(6)-Id, blaDHA-1, blaTEM-1, dfrA17, floR, mph(A), qnrB, sul1, sul2, tet(A*)	B1
		pR16.2845_95k	95,080	IncI1-I(Alpha)	*blaCMY-2*	
R17.0121	2017	R17.0121_chr	4,790,067			
		pR17.0121_164k	164,058	IncC	*aac(3)-IId, aph(3'')-Ib, aph(6)-Id, blaDHA-1, blaTEM-1, dfrA17, floR, mph(A), qnrB, sul1, sul2, tet(A*)	B1
R17.1451	2017	R17.1451_chr	4,790,379			
		pR17.1451_p159k	159,330	IncC	*aac(3)-IId, aadA2, aph(3'')-Ib, aph(3')-Ia, aph(6)-Id, blaDHA-1, blaTEM-1, dfrA14, floR, sul1, sul2, tet(A*)	A3
		pR17.1451_p102k	102,507	IncI1-I(Alpha)	*blaCMY-2, erm(B), sul2* (partial)*, tet(M*)	
R17.3494	2017	R17.3494_chr	4,790,068			
		pR17.3494_161k	161,012	IncC	*aac(3)-IId, aph(3'')-Ib, aph(6)-Id, blaDHA-1, blaTEM-1, dfrA17, floR, mph(A), qnrB, sul1, sul2, tet(A*)	B1
R18.0292	2018	R18.0292_chr	4,790,069			
		pR18.0292_200k	199,721	IncC	*aac(3)-IId, aadA2, aph(3'')-Ib, aph(3')-Ia, aph(6)-Id, blaDHA-1, blaTEM-1, floR, mph(A), qnrB, sul1, sul2, tet(A*)	A5
		pR18.0292_89k	88,662	IncI1-I(Alpha)	*erm(42*)	
R18.1932	2018	R18.1932_chr	4,790,068			
		pR18.1932_161k	161,012	IncC	*aac(3)-IId, aph(3'')-Ib, aph(6)-Id, blaDHA-1, blaTEM-1, dfrA17, floR, mph(A), qnrB, sul1, sul2, tet(A*)	B1
R19.1295	2019	R19.1295_chr	4,790,066			
		pR19.1295_161k	161,012	IncC	*aac(3)-IId, aph(3'')-Ib, aph(6)-Id, blaDHA-1, blaTEM-1, dfrA17, floR, mph(A), qnrB, sul1, sul2, tet(A*)	B1
R21.1488	2021	R21.1488_chr	4,790,067			
		pR21.1488_161k	161,022	IncC	*aac(3)-IId, aph(3'')-Ib, aph(6)-Id, blaDHA-1, blaTEM-1, dfrA17, floR, mph(A), qnrB, sul1, sul2, tet(A*)	B1
R22.1282	2022	R22.1282_chr	4,790,066			
		pR22.1282_161k	161,022	IncC	*aac(3)-IId, aph(3'')-Ib, aph(6)-Id, blaDHA-1, blaTEM-1, dfrA17, floR, mph(A), qnrB, sul1, sul2, tet(A*)	B1
R22.1933	2022	R22.1933_chr	4,790,067			
		pR22.1933_161k	161,022	IncC	*aac(3)-IId, aph(3'')-Ib, aph(6)-Id, blaDHA-1, blaTEM-1, dfrA17, floR, mph(A), qnrB, sul1, sul2, tet(A*)	B1
R23.0439	2023	R23.0439_chr	4,790,066			
		pR23.0439_162k	162,339	IncC	*aac(3)-IId, aph(3'')-Ib, aph(6)-Id, blaDHA-1, blaTEM-1, dfrA17, floR, mph(A), qnrB, sul1, sul2, tet(A*)	B1_v
		pR23.0439_104k	104,584	IncFIB(pHCM2)	*dfrA51*	
R23.0441	2023	R23.0441_chr	4,790,066			
		pR23.0441_162k	162,339	IncC	*aac(3)-IId, aph(3'')-Ib, aph(6)-Id, blaDHA-1, blaTEM-1, dfrA17, floR, mph(A), qnrB, sul1, sul2, tet(A*)	B1_v
		pR23.0441_104k	104,584	IncFIB(pHCM2)	*dfrA51*	
R23.0682	2023	R23.0682_chr	4,790,066			
		pR23.0682_162k	162,339	IncC	*aac(3)-IId, aph(3'')-Ib, aph(6)-Id, blaDHA-1, blaTEM-1, dfrA17, floR, mph(A), qnrB, sul1, sul2, tet(A*)	B1_v
		pR23.0682_104k	104,584	IncFIB(pHCM2)	*dfrA51*	
R23.1695	2023	R23.1695_chr	4,790,066			
		pR23.1695_162k	162,339	IncC	*aac(3)-IId, aph(3'')-Ib, aph(6)-Id, blaDHA-1, blaTEM-1, dfrA17, floR, mph(A), qnrB, sul1, sul2, tet(A*)	B1_v
		pR23.1695_104k	104,584	IncFIB(pHCM2)	*dfrA51*	
R23.2184	2023	R23.2184_chr	4,790,066			
		pR23.2184_162k	162,339	IncC	*aac(3)-IId, aph(3'')-Ib, aph(6)-Id, blaDHA-1, blaTEM-1, dfrA17, floR, mph(A), qnrB, sul1, sul2, tet(A*)	B1_v
		pR23.2184_104k	104,584	IncFIB(pHCM2)	*dfrA51*	
R24.0342	2024	R24.0342_chr	4,830,862			
		pR24.0342_162k	162,339	IncC	*aac(3)-IId, aph(3'')-Ib, aph(6)-Id, blaDHA-1, blaTEM-1, dfrA17, floR, mph(A), qnrB, sul1, sul2, tet(A*)	B1_v
		pR24.0342_104k	104,584	IncFIB(pHCM2)	*dfrA51*	

^*a*^ Only chromosomes and plasmids carrying ARGs are listed.

### MDR gene clusters in IncC plasmids

**Fig 2:**
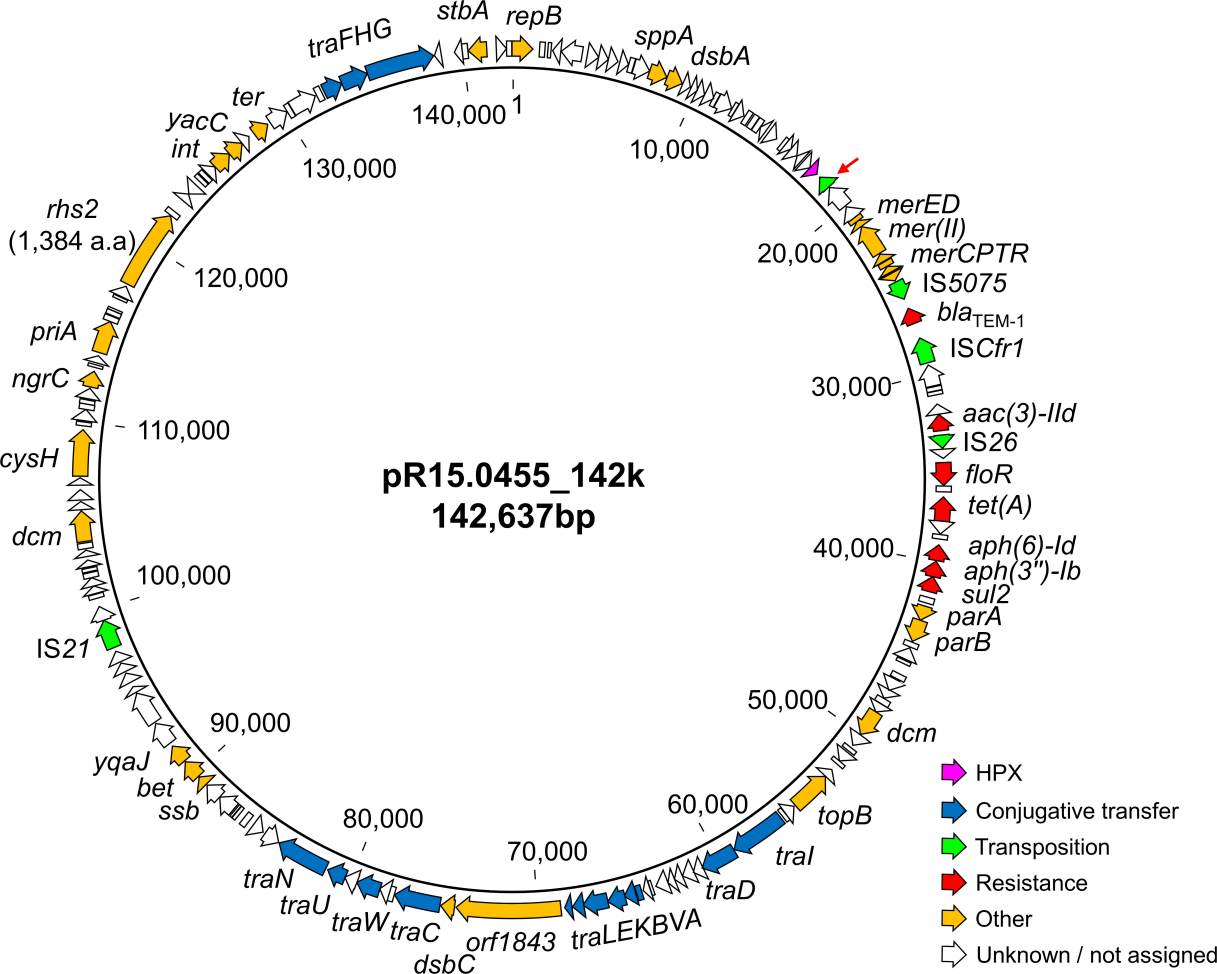
Genetic map of plasmid pR15.0455_142k, which contains the MDR gene cluster O and represents the conserved backbone shared among IncC plasmids carrying MDR gene clusters A1–A5, B, and B1_v. Most variants of the MDR gene cluster were generated through recombination-mediated integration of additional ARGs and accessory genes at the IS*26* adjacent to a 165-aa hypothetical protein (HPX), indicated by a red arrow. Functional gene categories are color-coded as shown in the legend.

Comparative analysis of IncC plasmids from MDR and AziR-XDR *S. Typhimurium* isolates identified seven distinct MDR gene clusters. A plasmid carrying the MDR gene cluster O likely represented the ancestral configuration, harboring seven ARGs, including *blaTEM-1*, *aac(3)-IId*, *floR*, *tet(A*), *aph(6)-Id*, *aph(3'')-Ib*, and *sul2* (Fig. 2). The segment containing *blaTEM-1 and aac(3)-IId* was flanked by IS*26* and observed in either orientation among the derived MDR gene clusters. The plasmid harboring MDR gene cluster O represented the conserved backbone from which plasmids carrying clusters A1–A5, B1, and B1_v were derived.

**Fig 3:**
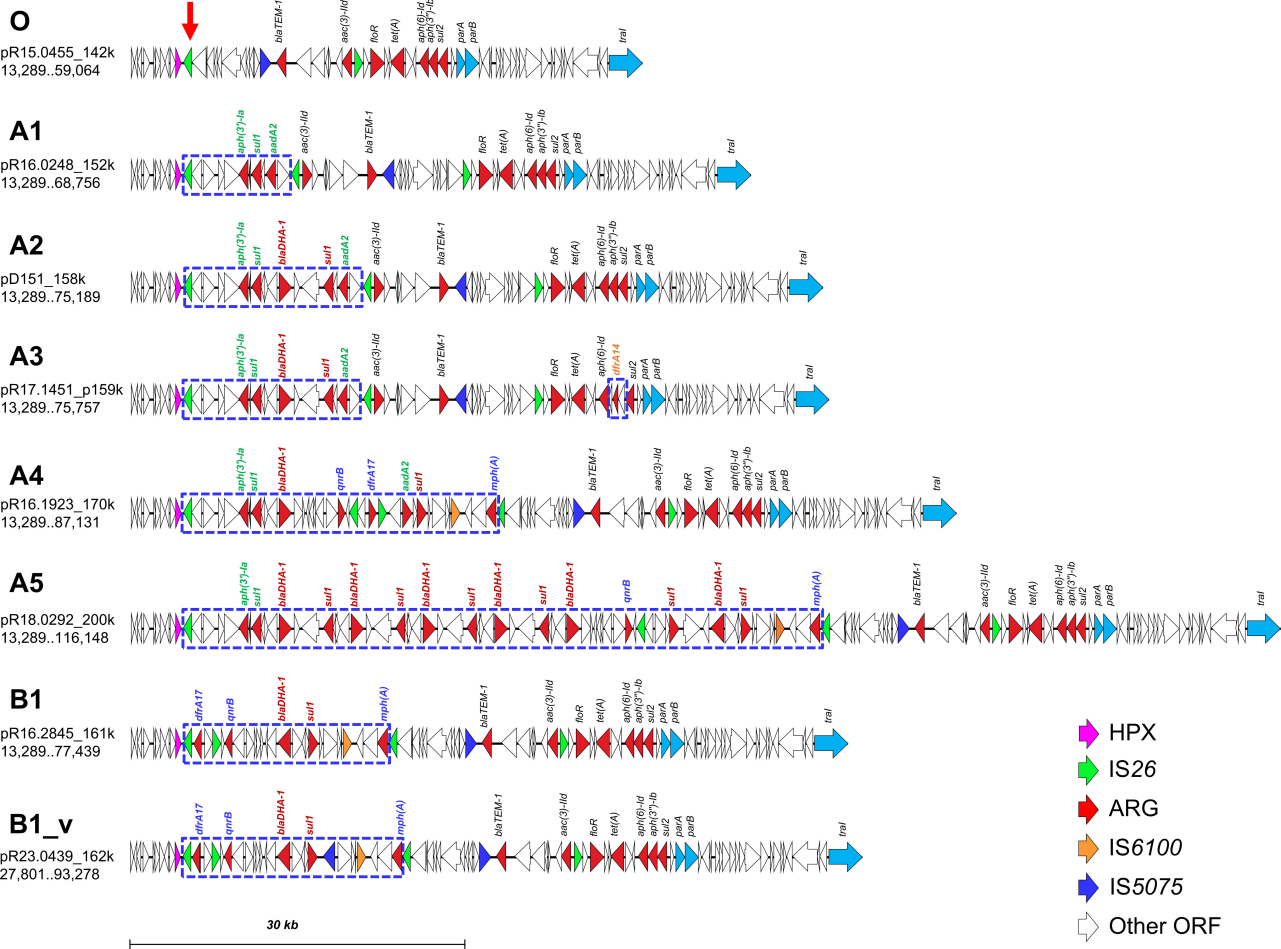
Comparative organization of MDR gene clusters in IncC plasmids from multidrug-resistant (MDR) and azithromycin-resistant, extensively drug-resistant (AziR-XDR) *Salmonella enterica* serovar Typhimurium isolates. Antimicrobial resistance genes (ARGs), insertion sequences (IS*26*, IS*5075*, IS*6100*), and other relevant genetic elements are color-coded. Open reading frames (ORFs) other than ARGs, the hypothetical protein (HPX), and IS elements are shown in white. The red arrowhead indicates the conserved IS*26* site mediating integration of additional ARGs and accessory genes. The azithromycin resistance gene *mph(A*) is located within an IS*6100*–IS*26*–flanked segment. ARG cluster patterns A4, B1, and B1_v were identified in plasmids from AziR-XDR isolates, whereas the remaining patterns were observed in plasmids from MDR isolates.

Among the MDR gene clusters, most variations resulted from the integration of additional ARGs and accessory genes through IS*26*-mediated homologous recombination at the IS*26* adjacent to a 165 amino-acid hypothetical protein, designated HPX (Fig. 3), as evidenced by the absence of 8-bp target site duplications at the IS*26* junctions. Compared with MDR gene cluster O, cluster A1 contained an inserted segment comprising IS*26*, *aph(3’)-Ia*, *sul1*, *aadA2,* and several accessory genes. Cluster A2 carried an additional segment containing *blaDHA-1*, *sul1*, and other accessory genes relative to A1, whereas cluster A3 was nearly identical to cluster A2 but had *dfrA14* in place of *aph(3’’)-Ib*. Cluster A4 acquired a segment with three additional ARGs, *qnrB*, *dfrA17*, and *mph(A*), relative to A2. Cluster A5, compared with cluster A4, contained five additional tandem copies of *blaDHA-1* and *sul1* but lacked *dfrA17* and *aadA2*. Cluster B1 lacked the *aph(3’)-Ia–sul1–aadA2* cassette found in clusters A1–A4 but included an insertion containing five ARGs, *dfrA17*, *qnrB*, *blaDHA-1*, *sul1*, and *mph(A*), at the HPX–IS*26* site. Cluster B1_v shared the same ARG composition as cluster B1 but contained an additional IS*5075* element.

Clusters A4, B1, and B1_v were exclusively observed in AziR-XDR isolates. Cluster B1_v emerged in 2023 and subsequently became the predominant type, whereas cluster A4 was infrequently detected. The azithromycin-resistance gene, *mph(A*), was arranged in the genetic structure of IS*26-mph(A)-mrx(A)-mphR(A*)-IS*6100*.

### Distribution of IS*26*-flanked ARG modules across plasmid backbones and species

To examine the distribution of the IS*26*-flanked 5-ARG segment harboring *dfrA17*, *qnrB*, *blaDHA-1*, *sul1*, and *mph(A*), identified in MDR gene cluster B, we compared the corresponding sequence region (18,007–37,086 nt of pR18.1932_161k; accession no. CP100733.1) against the NCBI database using BLAST. Among the 100 top hits, 37 plasmids and 2 chromosomes from 8 bacterial species carried the complete 5-ARG segment (Table S6). The species included *Citrobacter amalonaticus* (*n* = 1), *Enterobacter asburiae* (*n* = 1)*, Enterobacter hormaechei* (*n* = 3), *Escherichia coli* (*n* = 24), *Klebsiella pneumoniae* (*n* = 3), *Salmonella enterica* (*n* = 2), *Shigella flexneri* (*n* = 2), and *Shigella sonnei* (*n* = 3) (Table S7). Among the 37 plasmids, 33 carried an IncFII replicon or its variant IncFII(pRSB107), and 13 were hybrid plasmids with multiple replicons. Of the remaining four plasmids, three carried IncHI2–IncHI2A replicons and one carried IncHI1A(NDM-CIT)–IncHI1B(pNDM-CIT) replicons (Table S7). Both chromosomes also contained plasmid replicons, Col156–IncFII and IncQ1, respectively, indicating an association between the 5-ARG segment and IncFII plasmids. In addition, BLAST searches identified a related IS*26*-flanked four-ARG (4-ARG) segment carrying four ARGs, *qnrB*, *blaDHA-1*, *sul1*, and *mph(A*), but lacking the IS*26-dfrA17* element, in 37 additional plasmids and 3 chromosomes from six *Enterobacteriaceae* species (Table S8). Of these plasmids, 21 carried an IncR replicon, 12 had an IncF replicon or IncF-variants, 3 belonged to the IncHI family, and 1 was an IncC plasmid. Plasmids carrying either the IS*26*-flanked 5-ARG or 4-ARG segment were generally large (49–368 kb) and frequently exhibited multireplicon architectures. Collectively, these findings indicate that IS*26*-flanked ARG modules circulate widely across *Enterobacteriaceae*, with IncFII and IncR plasmids serving as the primary reservoirs. In *Salmonella*, these modules have been identified on IncC, IncFII, and IncHI2–IncHI2A–IncX1 plasmids (Tables S7 and S8). The presence of IS*26*-flanked 5-ARG and 4-ARG segments in multiple plasmid backbones highlights that *Salmonella* can acquire such resistance determinants from diverse sources, suggesting that horizontal transfer plays a key role in shaping the resistome of pathogenic *Salmonella*.

## DISCUSSION

Our findings demonstrate that AziR-XDR *S*. Typhimurium likely evolved locally from MDR ancestors through sequential IS26-mediated acquisitions on type 2 *IncC* plasmids. As illustrated in Fig. 3, AziR-XDR strains most likely evolved from a prototype plasmid with MDR gene cluster O, which harbored 7 ARGs conferring resistance to ampicillin, chloramphenicol, gentamicin, streptomycin, sulfonamides, and tetracycline but not to trimethoprim. The subsequent acquisition of *dfrA17*, *blaDHA-1*, *qnrB*, *sul1*, and *mph(A*), whether through stepwise accumulation (MDR gene cluster O → A1 → A2 → A4) or direct integration (MDR gene cluster B), conferred additional resistance to trimethoprim-sulfamethoxazole, fluoroquinolones, and third-generation cephalosporins, while also introducing azithromycin resistance, thereby meeting the definition of AziR-XDR.

Clinically, this is concerning because azithromycin and carbapenems remain critical therapeutic options for XDR *S*. Typhi and invasive NTS infections (4, 7, 12). The emergence and dissemination of AziR-XDR *S*. Typhimurium, thus, present a major challenge for patient management. Importantly, IncC plasmids are self-transmissible plasmids capable of mobilizing ARGs across diverse Proteobacteria hosts (13–15). Thus, their broad host range of conjugative transfer facilitates the spread of resistance, increasing the associated public health risk due to AziR-XDR phenotypes in *Salmonella*.

Among the MDR and AziR-XDR isolates examined, we identified seven distinct IncC-borne ARG architectures (MDR gene clusters O, A1–A5, B1, and B1_v) (Fig. 3). An IncC plasmid with MDR gene cluster O represents the ancestral configuration, whereas subsequent variants evolved through repeated IS*26*-mediated insertions, rearrangements, and deletions at the conserved HPX–IS*26* site. Consistent with our BLAST survey (Tables S7 and S8), the IS*26* flanked 5-ARG (*dfrA17*, *qnrB*, *blaDHA-1*, *sul1*, and *mph(A*) segment, and a cognate 4-ARG (*qnrB*, *blaDHA-1*, *sul1*, and *mph(A*)) segment, are widespread on large, often multireplicon plasmids, predominantly IncFII, IncHI, and IncR across many *Enterobacteriaceae* species, which likely serve as reservoirs from which IncC in *Salmonella* can acquire these ARG cassettes. Such remodeling also generated alternative configurations, exemplified by MDR gene cluster A5, which carries six tandem copies of a *sul1–blaDHA-1* cassette but lacks *dfrA17*. Although A5 is not AziR-XDR because trimethoprim resistance is absent, it retains *qnrB* and *mph(A*), sustaining fluoroquinolone and azithromycin resistance. These outcomes are consistent with the known activities of IS*26*, including the assembly and capture of multi-gene segments as pseudo-compound transposons and translocatable units, together with targeted conservative cointegration and tandem amplification, which provide a parsimonious route for cassette gain, loss, and expansion on the observed IncC plasmids (16). These interpretations are also consistent with Allain et al. (15), who demonstrated that IncC plasmids undergo extensive IS*26*-driven remodeling of antibiotic resistance islands, including cassette insertions, deletions, and amplifications at conserved junctions, and thereby generate diverse ARG architectures.

In Taiwan, XDR NTS has mainly been found in *S*. Anatum and *S*. Goldcoast. XDR *S*. Anatum was first identified in 2015, carrying 11 ARGs, *aadA2*, *blaDHA-1*, *dfrA23*, *floR*, *lnu(F*), *qnrB*4, *aph(3”)-Ib*, *aph(6)-Id*, *sul1*, *sul2*, and *tet(A*), on a 90-kb IncC plasmid; following the emergence of these XDR *S*. Anatum strains, the proportion of *S*. Anatum isolates increased sharply (13). XDR *S. Goldcoast* was first detected in 2017, and its prevalence increased rapidly thereafter. These strains commonly harbored 14 ARGs (*aac(3)-IId*, *aadA2*2, *aph(3’)-Ia*, *aph(6)-Id*, *arr-2*, *blaCTX-M-55*, *blaTEM-1B*, *dfrA14*, *floR*, *lnu(F), qnrS13*, *sul2*, *sul3*, and *tet(A*)), together with *ramAp*, an activator of efflux pump expression, on a large IncHI2 plasmid (17, 18). XDR *S*. Typhimurium strains were occasionally observed. AziR-XDR *S*. Typhimurium within the HC50_13 subclone was first identified in 2016, and its proportion markedly increased after 2021, accounting for 34.8% of *S*. Typhimurium isolates and 9.3% (152/1,635) of all *Salmonella* isolates in 2023 (Table S1). As IncC plasmids are well documented as self-transmissible elements capable of mobilizing ARGs across diverse bacterial hosts (13–15), the presence of AziR-XDR determinants on IncC plasmids in *S*. Typhimurium suggests that these strains may readily disseminate resistance determinants via horizontal transfer.

Notably, comparable trends have been documented in sub-Saharan Africa for invasive *S*. Typhimurium ST313. Van Puyvelde et al. reported the emergence of XDR *S*. Typhimurium ST313 in multiple countries (19, 20). In addition, pandrug-resistant (PDR) *S*. Typhimurium ST313 strains, functionally equivalent to the AziR-XDR phenotype described here, as they remained susceptible only to meropenem, were independently detected at three geographically distinct sites in the Democratic Republic of the Congo. These XDR and PDR ST313 isolates were strongly linked to IncHI2 and IncI1 plasmids (19), whereas in Taiwan, AziR-XDR *S*. Typhimurium strains predominantly harbor ARGs on IncC plasmids. Collectively, these observations highlight that distinct plasmid backbones can mediate the convergence of resistance determinants. Still, the public health implication remains the same: once established, AziR-XDR strains are primed for rapid regional and interregional dissemination, posing a serious global health threat.

Our analysis of 51 HC50_13 MDR and AziR-XDR isolates, recovered between 2012 and 2024 from humans, ducks, pigs, and chicken meat, revealed that these strains are highly related, all belonging to HC20_152604. The genomic differences among them were within 20 core genes, despite being collected over 13 years. According to the NCBI Pathogen Detection database, these isolates are assigned to SNP cluster PDS000042202. As of August 26, 2025, this cluster comprised 52 isolates, with only one strain (BioSample no. SAMN20181414) originating from Ontario, Canada, in 2015, while all others were from Taiwan. These findings indicate that the HC20_152604 (PDS000042202) clade has been predominantly circulating in Taiwan, where it has repeatedly acquired additional ARGs, ultimately giving rise to AziR-XDR strains resistant to the traditional first-line antimicrobials, fluoroquinolones, third-generation cephalosporins, and azithromycin.

Previous studies reported that the azithromycin resistance rate among human NTS isolates in Taiwan increased from 3.1% in 2017–2018 to 5.9% in 2021–2022 (1, 8). In the United States, the AST data indicated that the resistance rates were 0.4% in 2017–2018 and 1.6% in 2021–2022 (data obtained from the Centers for Disease Control and Prevention; https://www.cdc.gov/ncezid/dfwed/beam-dashboard.html/). In Europe, the rates were 0.5%, 0.6%, and 0.9% in 2021, 2022, and 2023, respectively (21–23). These findings indicate that azithromycin resistance in Taiwan NTS isolates is substantially higher than in the United States and Europe though an increasing trend is observed across all three regions.

In conclusion, although the HC50_13 subclone of *S*. Typhimurium is widely distributed, the MDR and AziR-XDR strains analyzed here fall within a distinct clade, HC20_152604 (SNP cluster PDS000042202). This clade has been detected almost exclusively in Taiwan, suggesting a local origin followed by repeated IS*26*-mediated acquisitions of resistance determinants on IncC plasmids. To date, there is no evidence of significant dissemination beyond Taiwan. The emergence of AziR-XDR strains, resistant to the traditional first-line antimicrobials, fluoroquinolones, third-generation cephalosporins, and azithromycin, poses a major clinical challenge by leaving very few therapeutic options available. To contain this clade, ongoing genomic surveillance, reinforced antimicrobial stewardship, and monitoring of animal and food reservoirs will be essential. Future studies should further investigate the evolutionary dynamics and ecological fitness of AziR-XDR strains, as well as their potential for international spread through trade, travel, or zoonotic transmission.

## MATERIALS AND METHODS

### Bacterial isolates and epidemiologic metadata

*Salmonella* isolates were obtained from human salmonellosis cases through PulseNet Taiwan, a national molecular surveillance network established in 2004. The system collects isolates via collaborating hospitals across Taiwan. The surveillance was approved by the Institutional Review Board of the Taiwan Centers for Disease Control (Taiwan CDC). Species identification was confirmed using the MALDI Biotyper system (Bruker Corp., USA). Genotyping was conducted using the standardized PulseNet pulsed-field gel electrophoresis (PFGE) protocol (24), and serotypes were assigned by comparing PFGE patterns with those in the Taiwan CDC *Salmonella* PFGE database (25). This study included *S*. Typhimurium isolates collected between 2004 and 2024. Epidemiologic metadata, including year of isolation, patient age, sex, and infection site, were provided by the submitting hospitals or obtained from the Laboratory Automated Reporting System (LARS) established by the Taiwan CDC. Isolates recovered from blood, cerebrospinal fluid, or other non-intestinal sites were classified as extraintestinal, whereas those from stool, anal swabs, or rectal swabs were classified as intestinal.

### Antimicrobial susceptibility testing

AST was performed using the EUVSEC3 Sensititre MIC panel (TREK Diagnostic Systems Ltd., West Essex, England), which contains 15 antimicrobial agents selected according to the European Union protocol for antimicrobial resistance (AMR) monitoring in *Salmonella* spp. (22). The antimicrobial agents were chosen for their clinical relevance and public health importance. Minimum inhibitory concentration (MIC) results were interpreted using Clinical and Laboratory Standards Institute (CLSI) breakpoints for *Enterobacterales* (26), covering amikacin, ampicillin, azithromycin, cefotaxime, ceftazidime, chloramphenicol, ciprofloxacin, colistin, gentamicin, meropenem, nalidixic acid, sulfamethoxazole, tetracycline, and trimethoprim. For tigecycline, no interpretative criteria are provided by CLSI, and the European Committee on Antimicrobial Susceptibility Testing (EUCAST) has not established an epidemiological cutoff value (ECOFF) for *Salmonella*; therefore, we adopted the EU surveillance system’s resistance breakpoint of > 0.5 mg/L (22). For ciprofloxacin, MIC values were interpreted using CLSI criteria, where isolates were considered susceptible at MIC < 0.125 mg/L, intermediate (reduced susceptible) at MIC = 0.125–0.5 mg/L, and resistant at MIC ≥ 1 mg/L.

MDR strains were defined as those resistant to at least three different classes of antimicrobials. XDR strains were defined, following the *S. Typhi* definition, as those resistant to the three traditional first-line antimicrobials (ampicillin, chloramphenicol, and trimethoprim-sulfamethoxazole), plus a fluoroquinolone (e.g., ciprofloxacin) and a third-generation cephalosporin (e.g., cefotaxime or ceftriaxone) (27). Given that even reduced ciprofloxacin susceptibility (MIC 0.125–0.5 mg/L) can significantly compromise clinical outcomes, including prolonged fever clearance time and treatment failure (28, 29), we further classified strains with reduced ciprofloxacin susceptibility, together with resistance to the first-line antimicrobials and either cefotaxime or ceftazidime, as XDR. AziR-XDR strains were defined as XDR strains that also exhibited resistance to azithromycin.

### Whole-genome sequencing and analysis

Sixty *S. Typhimurium* isolates were selected for WGS, including 45 MDR and 15 AziR-XDR strains, recovered between 2007 and 2024 from humans (*n* = 54), ducks (*n* = 4), pig (*n* = 1), and chicken meat (*n* = 1) (Table S3). All 60 isolates were initially sequenced using Illumina short-read technology. WGS of 53 isolates was performed by the Taiwan CDC, and 7 isolates by Dr. Achtman’s team at the Wellcome Sanger Institute and the University of Warwick. Among these, 25 selected isolates were further sequenced using the Oxford Nanopore Technologies (ONT) platform to generate long reads for complete genome assembly. Genomic DNA was extracted using the DNeasy Blood & Tissue Kit (cat. #69506, Qiagen, Hilden, Germany) according to the manufacturer’s instructions. Short-read libraries were prepared using the Illumina DNA Prep kit (cat. IL20018705, Illumina Inc., San Diego, CA, USA), which includes tagmentation of genomic DNA, bead-based cleanup, PCR amplification with adapters, and final purification. Illumina sequencing was performed on the MiSeq platform (Illumina Inc.), using a 2 × 300 bp paired-end configuration, and all isolates achieved a depth of coverage > 30 ×. Raw reads were processed using fastp (https://github.com/OpenGene/fastp) for quality filtering and adapter removal, and *de novo* assemblies were processed using SPAdes v3.15.3 (30). Low-quality contigs were excluded based on length (<200 bp), read coverage (<2×), or homopolymer composition. Assemblies were analyzed using AMRFinderPlus (31) to identify antimicrobial resistance determinants, SISTR (https://github.com/phac-nml/sistr_cmd) for *in silico* serotype prediction, and PlasmidFinder (http://www.genomicepidemiology.org) to identify plasmid incompatibility (replicon) types. IncC plasmid typing was performed according to the classification scheme described by Ambrose et al. (32) based on sequence analysis of specific marker genes (*rhs1/rhs2* and *orf1832/1847*) in comparison with the reference IncC plasmids pR148 (type 1, GenBank accession no. JX141473) and R55 (type 2, GenBank accession no. JQ010984). For the selected 25 isolates, long-read sequencing was performed on the MinION platform (Oxford Nanopore Technologies, Oxford, UK) using R10.4.1 flow cells. Raw signal data (POD5) were basecalled with Dorado v0.5.0 using the SUP4.3+ mode to generate FASTQ reads. Long-read sequences of each isolate were assembled using Flye v2.9.6 (https://github.com/mikolmogorov/Flye). The assembled circular sequences were reoriented using dnaapler v1.2.0 (https://github.com/gbouras13/dnaapler), and polished using Medaka v2.0.1 (https://github.com/nanoporetech/medaka).

Illumina reads were uploaded to EnteroBase (https://enterobase.warwick.ac.uk/) to obtain HierCC clustering assignments at multiple levels. In this framework, isolates differing by >100 core genes were considered distinct clones (HC100), those differing by ≤50 core genes as subclones (HC50), and those differing by ≤20 core genes as clades (HC20), representing groups of very high genetic similarity. All WGS data are publicly available in the NCBI database, and the corresponding accession numbers are listed in Table S3. To assess genomic relatedness with publicly available genomes, we used the NCBI Pathogen Detection system (https://www.ncbi.nlm.nih.gov/pathogens/), which automatically assigns isolates to SNP clusters. These SNP clusters provide single-level groupings of closely related isolates that are useful for broad genomic comparisons but lack the hierarchical resolution of HierCC.

For cross-clone comparisons of infection type and demographic distribution, HC100 clones were determined for *S. Typhimurium* isolates obtained between 2004 and 2024, using PFGE clustering with representative WGS-based HierCC assignments as described previously (11).

### Comparative analysis of IS*26*-flanked antimicrobial resistance gene segments

The IS*26*-flanked segment containing *dfrA17*, *qnrB*, *blaDHA-1*, *sul1*, and *mph(A*), named as 5-ARG segment, in pR18.1932_161k (GenBank accession no. CP100733.1; positions 18,007–37,086 nt) was queried against the NCBI nucleotide collection using BLAST. The sequences of the top 100 BLAST hits were downloaded, and plasmid replicons and ARGs were identified using PlasmidFinder and AMRFinderPlus.

### Statistical analysis

Chi-square tests were performed to compare extraintestinal infection rates and demographic (sex and age groups) distributions between major *S*. Typhimurium clones and between AziR-XDR and non-AziR-XDR isolates within the HC100_13 clone. A *P*-value of <0.05 was considered statistically significant.

## Data Availability

Genomic sequences of the 60 *S*. Typhimurium isolates analyzed in this study are available in the NCBI database under BioProjects PRJNA478278 and PRJEB20997. Accession numbers for individual isolates are provided in Table S3.

## SUPPLEMENTAL MATERIAL

Supplemental material aac.01334-25-s0001.xlsx Tables S1 to S8.

ASM does not own the copyrights to Supplemental Material that may be linked to, or accessed through, an article. The authors have granted ASM a non-exclusive, world-wide license to publish the Supplemental Material files. Please contact the corresponding author directly for reuse.
